# Changes in Major Retinal Blood Vessel Position Outside the Optic Nerve Head in Glaucomatous Eyes

**DOI:** 10.18502/jovr.v20.15461

**Published:** 2025-07-10

**Authors:** Zahra Karjou, Shahin Yazdani, Behrouz Alizadeh Savareh, Bahareh Kheiri, Fatemeh Radinmehr

**Affiliations:** ^1^Ophthalmic Research Center, Research Institute for Ophthalmology and Vision Science, Shahid Beheshti University of Medical Sciences, Tehran, Iran; ^2^Ocular Tissue Engineering Research Center, Research Institute for Ophthalmology and Vision Science, Shahid Beheshti University of Medical Sciences, Tehran, Iran; ^3^National Agency for Strategic Research in Medical Education, Tehran, Iran; ^4^Optometrist, Private Practice, Tehran, Iran

**Keywords:** Glaucoma, Optic Nerve Head, Vascular Displacement

## Abstract

**Purpose:**

Vascular changes along with loss of the neural rim at the optic nerve head (ONH) are established hallmarks of glaucomatous optic neuropathy. The current study investigates changes in the position of major retinal vessels outside the ONH in eyes with definite or suspected glaucoma and reports its clinical associations.

**Methods:**

This retrospective case–control study was conducted on a dataset of 2390 patients with definite or suspected glaucoma and serial photographic documentation from 2015 to 2022. Images were overlaid chronologically and examined for vascular displacement (VD) outside the margin of the ONH up to one disc diameter using the built-in fundus camera software; in the case of VD detection, the change was verified using MATLAB software. The amount of VD was measured in pixels and expressed in a unitless arbitrary ratio derived from the amount of VD in pixels divided by the largest optic disc diameter in pixels. During the study period, a small number of eyes showed evidence of VD, which made up the case group; eyes without evidence of VD from the same dataset were chosen as controls.

**Results:**

A total of 23 eyes demonstrated VD, and 60 eyes with no evidence of VD were selected as controls. The mean amount of VD was 0.15 ± 0.01 in case eyes compared to 0.01 ± 0.01 in control eyes (*P* < 0.001). Definite glaucomatous damage was observed in 20 (87%) eyes in the case group compared to 35 (58.3%) eyes in the control group (*P* = 0.014). The best-corrected visual acuity in eyes with VD, both at baseline and at the final visit, was significantly worse than in controls (*P *= 0.018 and *P* = 0.032, respectively). Eyes with VD had greater cupping both at baseline (*P* = 0.025) and at the final examination (*P* = 0.04). During the study period, 16 (69.6%) eyes with VD and 12 (20%) control eyes required glaucoma surgery (*P* = 0.001). Patients with VD also showed a statistical trend toward being younger (mean age, 54.5 ± 16.5 vs 61.3 ± 15.5 years, *P* = 0.088).

**Conclusion:**

VD outside the ONH may occur in eyes with glaucoma and is associated with factors reflecting more significant glaucomatous damage. Eyes with VD outside the ONH have lower visual acuity, greater cupping, and require glaucoma surgery more often, indicating more significant glaucoma severity or progression.

##  INTRODUCTION

The central retinal artery (CRA) enters the optic nerve, passes through the lamina cribrosa, and branches off to supply the inner retina.^[1]^ Large retinal vessels are located in the innermost layers of the retina close to the internal limiting membrane, and their walls are in close contact with glial cells, especially astrocytes.^[2]^ Several studies have reported vascular changes and displacement at the optic nerve head (ONH) associated with glaucomatous optic neuropathy (GON). Varma et al demonstrated nasalization of the retinal blood vessels at the ONH for the first time.^[3]^ Subsequently, various studies have shown that along with ONH changes due to glaucoma, vessels at the optic nerve undergo positional changes.^[4,5]^ In 2015, Fuente et al proposed a method that can be used to detect glaucoma suspects by measuring the position of vessels at the ONH.^[6]^ In addition to glaucoma patients, positional shift of the central vessel trunk in the ONH has been reported with increasing axial length in myopic children in a study by Kyoung Min Lee et al, although no change was observed in the vascular arcades.^[7]^ Many glaucoma imaging devices employ vascular position to register and match images from one examination to the next.^[4,8,9,10]^ Therefore, displacement of retinal vessels is also essential in this regard.

The rationale behind the current study was an incidental observation. As part of a comprehensive glaucoma management scheme, we perform serial fundus photography for patients with definite and suspected glaucoma. Over a course of seven years, after comparing fundus images at different time points, we encountered instances when the vascular tree of fundus images failed to superimpose completely, suggesting displacement in certain segments of retinal vessels. These vascular changes were observed beyond the boundaries of the ONH, where vascular changes are an expected sign of glaucomatous damage. This led to a complete re-evaluation of our photographic database to explore this phenomenon and its significance and determine possible clinical correlations.

In this report, we discuss changes in the position of major retinal vessels outside the ONH over time and their clinical associations.

##  METHODS

In this retrospective study, all patients with confirmed or suspected glaucoma who visited the private practice of one of the authors from 2015 to 2022 and underwent serial photography of the ONH were enrolled. The study adhered to the tenets of the Declaration of Helsinki and was approved by the Research Institute for Ophthalmology and Vision Science. Regarding the anonymous use of existing data from patients who had previously consented to image acquisition, and the fact that no breach of patient privacy occurred and no further diagnostic or therapeutic procedures were performed for any subject, informed consent was not necessary for this study. Glaucoma is marked by a common feature of optic nerve degeneration and loss of retinal ganglion cells (RGCs). A glaucoma suspect is defined as someone who exhibits one or more clinical features and/or risk factors that increase the likelihood of developing glaucoma-related optic neuropathy (GON) and visual impairment in the future but does not meet the diagnostic criteria for functional and structural damage associated with GON. While patients with vascular displacement (VD) outside the ONH made up the case group, the control group was randomly selected from patients with no vascular changes during the same time period and with similar follow-up duration. We used a simple randomization technique to select control subjects. Utilizing a random number generator, we generated 60 unique random numbers within the range of the entire cohort.

Exclusion criteria included any ocular conditions that prevented the acquisition of high-quality images (e.g., nystagmus, media opacity, and poor fixation), disorders of the vitreoretinal interface, retinovascular disorders such as diabetic retinopathy and retinal vascular accidents, high myopia or hyperopia, severe chorioretinal atrophy, and ONH anomalies such as tilted discs, optic nerve hypoplasia, megalopapilla, and ONH drusen.

All subjects underwent serial fundus photography (Canon CR2 AF Stereoscopic Fundus Camera, Canon USA Inc., Melville, NY, USA) conducted by one experienced examiner. The first fundus photograph served as the baseline for all subjects, and the subsequent series of images were superimposed on the initial image using the built-in software. Serial images were examined for possible displacement of retinal vessels outside the ONH. To account for the field of view of the fundus camera and to improve the accuracy of image matching, a region of interest one disc diameter around the ONH was selected for the study.

**Figure 1:**
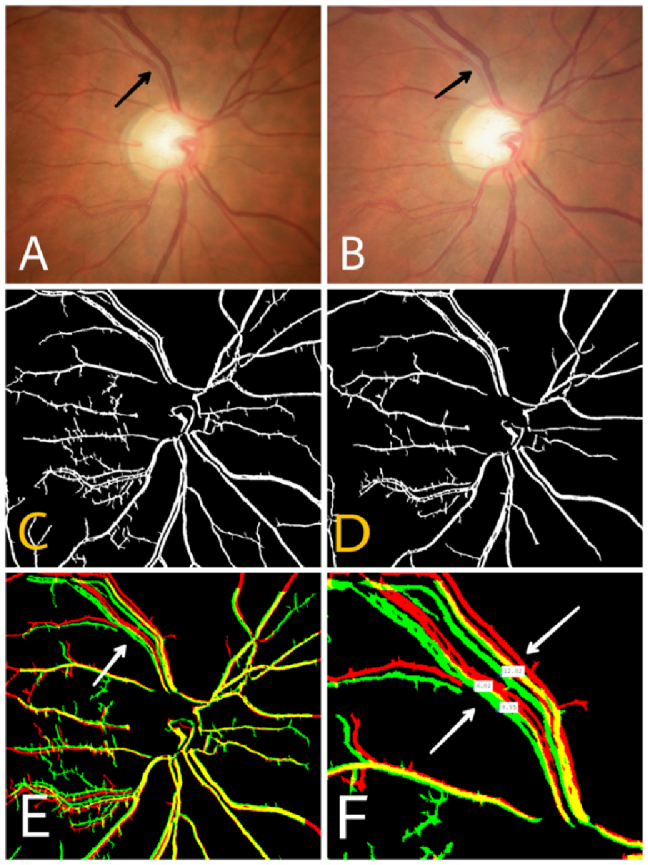
(A & B) Fundus photographs of a representative case of significant vascular displacement; the displaced vessel is marked with an arrow. (C & D) Vessel segmentation of previous images. (E) In the merged superimposed image, perfectly matching vessels are shown in yellow, while displaced vessels are depicted in green and red (arrow). In this patient, the vessels inside the optic nerve head remained unchanged while displacement occurred outside the ONH. (F) High-magnification image of the displaced vessels denoting the amount of displacement in pixels.

**Figure 2:**
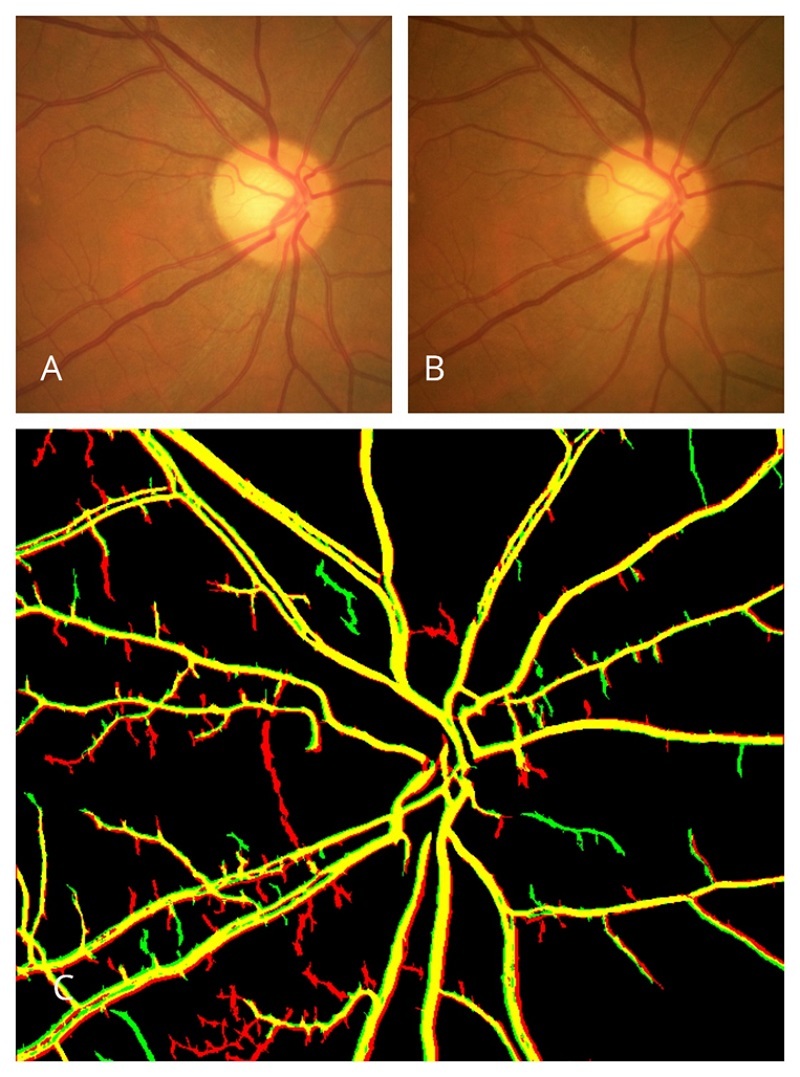
(A & B) Fundus photographs at baseline and last visit, respectively, in a representative eye from the control group. (C) The combined superimposed image created by the software shows no vascular displacement, as indicated by the yellow-colored vessels.

In the next stage, an independent examiner verified images from eyes with VD using MATLAB software. In a multistage procedure, image processing and correction were performed. This included vessel extraction and segmentation, correction for X–Y scale, image rotation, and correlation. Using this multistage approach, all properties of the two images (horizontal and vertical displacement, angular rotation, and scale) were evaluated and corrected if necessary, and the best-matched pair of images was selected for final comparison. In this manner, the most important confounding factors—specifically, the change in photography angle and eye rotation—were eliminated in the final adjusted image. Eventually, the major retinal vessels were superimposed; in this final combined image, perfectly aligned vessels were shown in yellow, while displaced vessels were shown in red on the first image and green on the subsequent image. The amount of VD was expressed in an arbitrary unit-less number derived from the ratio of VD in pixels to the largest diameter of the ONH of the same eye in pixels [Figures 1 & 2]. In this study, we defined significant VD as follows: when the displacement exceeded the diameter of the vessel, that is, we observed an empty space between the two vessels after the image overlap, we considered the displacement significant. There is no threshold for VD since this finding has not been previously addressed.

Patient records were reviewed for demographic information (age, sex, and history of systemic disease), history of ocular surgery, components of a detailed ocular examination (best-corrected visual acuity [BCVA] and vertical cup-to-disc ratio [VCDR]), and pathological findings on anterior segment examination or ONH and retina, at baseline and at the time of the last photography.

To describe data, mean, standard deviation, median, range, frequency, and percentage values were used. *T*-test, chi-square, and Fisher's exact tests were used to compare the parameters between the study groups. A paired *t*-test was used to compare the results within the groups. Statistical analysis was performed using SPSS 25.0 software package (IBM Corp., New York, NY, USA), and *P*-values < 0.05 were considered statistically significant.

**Table 1:** Demographic data of patients in the case and control groups

**Factors**		**Total**	**Study groups**		***P*** **-value**
			**Cases**	**Controls**	
Age	Mean ± SD	59.4 ± 16.3	54.5 ± 16.5	61.3 ± 15.9	0.088
	Median (range)	60 (14 to 91)	58 (14 to 74)	62 (28 to 91)	
Gender	Male	27 (32.5%)	6 (26.1%)	21 (35.0%)	0.602
	Female	56 (67.5%)	17 (73.9%)	39 (65.0%)	
Laterality	Right eye	47 (56.6%)	11 (47.8%)	36 (60.0%)	0.317
	Left eye	36 (43.4%)	12 (52.2%)	24 (40.0%)	
Diabetes	Yes	6 (7.2%)	3 (13.0%)	3 (5.0%)	0.340
	No	77 (92.8%)	20 (87.0%)	57 (95.0%)	
Hypertension	Yes	28 (33.7%)	7 (30.4%)	21 (35.0%)	0.694
	No	55 (66.3%)	16 (69.6%)	39 (65.0%)	

##  RESULTS

Records of 2390 patients with definite or suspected glaucoma and photographic documentation of the ONH were screened. Of these, 1393 eyes with a minimum of two high-quality fundus images at least one year apart were selected for the study. Using the built-in fundus camera software, significant VD was initially detected in 30 eyes. However, after image processing and correction with MATLAB software, the displacement was not confirmed in 7 eyes, resulting in their exclusion. Eventually, the case group comprised 23 eyes from 23 patients with confirmed vascular disease (VD) outside the optic nerve head (ONH) as identified by both imaging comparison modalities. For controls, sixty eyes from 60 subjects with a similar follow-up duration but no detectable VD outside the ONH by both modalities were selected from the same database.

The mean follow-up duration was 3.73 ± 0.7 years for cases and 3.83 ± 0.6 years for controls. The study groups had no significant differences regarding gender, systemic conditions, and eye laterality. Patients in the case group were younger than the controls (mean age, 54.5 ± 16.5 vs 61.3 ± 15.5 years, respectively). However, this difference only showed a borderline statistical significance (*P* = 0.088) [Table 1].

Of the 23 eyes with VD outside the ONH, 12 also had vascular changes at the ONH, whereas 11 eyes demonstrated VD only outside the ONH. The mean amount of VD was 0.15 ± 0.01 in cases and 0.01 ± 0.01 in controls (*P* < 0.001). A diagnosis of glaucoma was significantly more prevalent in eyes with VD; definite GON was present in 20 eyes (87%) of the case group compared to 35 eyes (58.3%) of the control group (*P* = 0.014) [Table 2].

The BCVA of eyes with VD was significantly worse than that of control eyes, both at baseline and at the final follow-up (P = 0.032 and P = 0.018, respectively). Within the case group, the visual acuity of eyes with VD was comparable to that of the fellow eyes of the same patients at baseline (P = 0.137); however, at the final follow-up, eyes with VD had significantly worse visual acuity than their fellow eyes (P = 0.031). During the study period, visual acuity showed significant deterioration in eyes with VD (P = 0.001) compared to that of fellow eyes of the same patients (P = 0.286) and control eyes (P = 0.50) [Table 3].

The mean VCDR was significantly higher in eyes with VD compared to controls at both baseline (0.73 ± 0.24 vs 0.61 ± 0.20, *P* = 0.025) and at the final follow-up (0.72 ± 0.24 vs 0.60 ± 0.26, *P* = 0.040). However, the ONH cupping in the fellow eyes of the case group was comparable to control eyes at both baseline and final follow-up (*P* = 0.279 and 0.257, respectively). In the case group, VCDR was significantly greater in eyes with VD compared to fellow eyes, both at baseline and at the final follow-up (*P* = 0.013 and *P* = 0.023, respectively) [Table 3]. Cupping remained stable throughout the follow-up period in all study groups.

Surgical history was comparable between cases and controls (*P* = 0.243). However, during the study period, 16 eyes (69.6%) with VD required some form of glaucoma surgery, whereas only 12 patients (20%) in the control group underwent surgical intervention for glaucoma (*P* = 0.001). In the case group, the need for glaucoma surgery was significantly higher in eyes with VD compared to fellow eyes (*P* = 0.029). The rate of glaucoma surgery in the fellow eyes of the case group was comparable to that of the control group (*P* = 0.778). Table 5 provides details of prior surgeries and interventions performed during the study period.

**Table 2:** Types of glaucoma in the study groups

**Factors**		**Total**	**Cases**	**Controls**	***P*** **-value**
Glaucoma	Yes	55 (66.3%)	20 (87.0%)	35 (58.3%)	0.014
	No	28 (33.7%)	3 (13.0%)	25 (41.7%)	
Primary open-angle glaucoma		18 (32.7%)	8 (40.0%)	10 (28.6%)	0.034
Primary angle closure glaucoma		28 (50.9%)	6 (30.0%)	22 (62.9%)	
Other categories of glaucoma		9 (16.4%)	6 (30.0%)	3 (8.6%)	

**Table 3:** Best-corrected visual acuity (logMAR) during the study period

**BCVA**	**Study groups**		***P*** **-value**
	**CasesMean ± SDMedian (Range)**	**ControlsMean ± SDMedian (Range)**	
Baseline	0.12 ± 0.22	0.04 ± 0.11	0.032
Index eye	0 (0 to 0.7)	0 (0 to 0.52)	
Baseline	0.05 ± 0.08	0.21 ± 0.60	0.290
Fellow eye	0 (0 to 0.30)	0 (0 to 2.70)	
*P*-value	0.137	0.036	
Final follow-up	0.16 ± 0.22	0.05 ± 0.15	0.018
Index eye	0.05 (0 to 0.7)	0 (0 to 1)	
Final follow-up	0.04 ± 0.06	0.21 ± 0.6	0.202
Fellow eye	0 (0 to 0.22)	0 (0 to 2.70)	
*P*-value	0.031	0.053	
Change (Baseline to final)Index eye	0.001	0.501	
Change (Baseline to final) Fellow eye	0.286	0.857	

**Table 4:** Vertical cup-to-disc ratio (VCDR) in the study groups

**VCDR**	**Study groups**		***P*** **-value**
	**CasesMean ± SDMedian (Range)**	**ControlsMean ± SDMedian (Range)**	
Baseline	0.73 ± 0.24	0.61 ± 0.20	0.025
Index eye	0.80 (0.10 to 1)	0.65 (0.10 to 0.90)	
Baseline	0.58 ± 0.22	0.64 ± 0.22	0.279
Fellow eye	0.6 (0.10 to 1)	0.65 (0.20 to 1)	
*P*-value	0.013	0.283	
Final follow-up	0.72 ± 0.24	0.60 ± 0.26	0.040
Index eye	0.8 (0.1 to 1)	0.65 (0.65 to 0.90)	
Final follow-up	0.60 ± 0.22	0.65 ± 0.21	0.257
Fellow eye	0.60 (0.10 to 1)	0.65 (0.20 to 1)	
*P*-value	0.023	0.059	
Change (Baseline to final)Index eye	0.726	0.381	
Change (Baseline to final) Fellow eye	0.328	0.108	

**Table 5:** Surgical procedures performed during the study period and past surgical history

**Factors**		**Study groups**		***P*** **-value**
		**Cases**	**Controls**	
SurgeryIndex eye	Glaucoma	12 (52.2%)	11 (18.3%)	0.001
	Cataract	1 (4.3%)	1 (1.6%)	
	Other	0 (0.0%)	1 (1.7%)	
	Cat + Glaucoma	4 (17.4%)	1 (1.7%)	
	No Surgery	6 (26.1%)	46 (76.6%)	
SurgeryFellow eye	Glaucoma	5 (21.7%)	14 (23.3%)	0.778
	Cataract	3 (13.0%)	1 (1.6%)	
	Other	0 (0.0%)	1 (1.7%)	
	Cat + Glaucoma	0 (0.0%)	3 (5.0%)	
	No Surgery	15 (65.2%)	41 (68.3%)	
*P*-value		0.029	> 0.999	
Status post	Glaucoma	3 (13.0%)	11 (18.3%)	0.243
	Cataract	3 (13.0%)	5 (8.3%)	
	Other	3 (13.0%)	1 (1.7%)	
	Cat + Glaucoma	1 (4.3%)	4 (6.7%)	
	No Surgery	13 (56.5%)	39 (65.0%)	

##  DISCUSSION 

Extensive literature exists regarding vascular changes at the ONH associated with GON.^[3][4,5][6][11][12][13][14][15][16]^ However, the current study is the first to report changes in major retinal blood vessel position outside the optic disc in eyes with glaucoma. Using serial fundus photography, we observed a previously unrecognized pattern of VD in major retinal vessels in a minority of eyes with suspected or definite glaucoma.

We employed two different image comparison/processing modalities to evaluate, verify, and quantify VD, and only included eyes in which both modalities confirmed the shift in vessel position. Initial screening was conducted using the built-in software provided by the fundus camera manufacturer. All eyes with suspected changes were further evaluated using more sophisticated image processing algorithms to rule out artifactual changes due to factors such as X–Y magnification, angular rotation, photography angle, and scale shift. We utilized adaptive histogram equalization, an adaptive median filter, and a Gaussian filter for image processing. This improved the quality of the input fundus images and eliminated unwanted noise, thereby enhancing the accuracy of the matching algorithm.^[17]^

Based on our observations, VD was significantly associated with factors indicating more severe or progressive glaucomatous damage. Eyes with VD had a higher rate of definitively diagnosed glaucoma, lower visual acuity, and a higher VCDR; these eyes also required glaucoma surgery more frequently than the control group during the study period, despite comparable rates of prior surgical procedures. These findings imply that the gradual shift in retinal vasculature is a slowly progressive phenomenon, probably associated with progressive GON. Even in the same individual, fellow eye comparison within the case group revealed that the eye with significant VD had more severe damage, determined by higher VCDR, lower visual acuity, and greater need for glaucoma surgery.

Varma et al evaluated changes in blood vessels at the ONH and concluded that vascular shifts occur in eyes with more severe glaucoma, younger subjects, and patients requiring more surgical interventions. They suggested that these changes can be used to detect glaucoma progression.^[3]^ It has been previously shown that RNFL changes and reversal of optic disc cupping occur more frequently in younger patients.^[18]^ Furthermore, in an animal study on monkeys, axonal damage due to elevated intraocular pressure was more pronounced in younger eyes. Consistent with the studies suggesting greater tissue plasticity in younger eyes,^[19]^ we observed that patients in our case group were younger than the controls. However, the difference only displayed a borderline trend.

Several investigations evaluating optic nerve vascular changes associated with glaucoma suggest that these changes are likely due to cup excavation or RNFL remodeling.^[4,6,20,21,22,23,24,25,26]^ In our study, 47.8% of eyes with VD showed changes only outside the ONH, while vessels at the optic nerve remained unchanged over time. This may imply that factors other than lamina cribrosa alterations or ONH remodeling are involved in the vascular changes associated with progressive glaucoma.

The patients studied herein were diagnosed and monitored by a single glaucoma sub-specialist using the same diagnostic and therapeutic approach, providing good internal validity for the study. This report also highlights the importance of fundus photography in managing glaucoma patients as an objective imaging modality that may provide additional data to optical coherence tomography (OCT) or visual fields (VF).

The limitations and drawbacks of our study include a lack of correlation with conventional structure-function tests using OCT and VF, which can determine whether structural and/or functional defects are related to the observed VD. Such correlations are the focus of an ongoing study.

In summary, our findings indicate an association between glaucoma progression and VD outside the ONH. In nearly half of these eyes, VDs were confined to the peripapillary area, while the remaining eyes showed vascular changes both at the ONH and in the peripapillary area. Eyes showing such changes had lower visual acuity, higher VCDR, and required more surgical interventions for glaucoma than a matched control group, indicating that glaucoma is more advanced/progressive in these patients. Significant differences in these factors were observed both when compared to control eyes of patients with no detectable vascular changes and fellow eyes of subjects demonstrating VD in one eye.

##  Financial Support and Sponsorship

None.

##  Conflicts of Interest

None.
